# Societal costs and health related quality of life in adult atopic dermatitis

**DOI:** 10.1186/s12913-023-09840-7

**Published:** 2023-08-14

**Authors:** Zsuzsanna Beretzky, Kamilla Koszorú, Fanni Rencz, Krisztina Hajdu, Júlia Borza, Katalin Bodai, Xu Feifei, Andrea Szegedi, Miklós Sárdy, Valentin Brodszky

**Affiliations:** 1 Department of Health Policy, Corvinus University of Budapest, 8 Fővám tér, Budapest, H-1093 Hungary; 2 Department of Dermatology, Venereology, and Dermatooncology, Faculty of Medicine, Semmelweis University, Budapest, Hungary; 3 Department of Dermatology, Faculty of Medicine, University of Debrecen, Debrecen, Hungary; 4 Centre of Excellence MTA and ELKH-DE Allergology Research Group, Debrecen, Hungary; 5 Saint Martin Outpatient Center, Pannonhalma, Hungary

**Keywords:** Atopic dermatitis, Dermatology, Cost, Disease burden, DLQI, EQ-5D

## Abstract

**Background:**

Cost-of-illness studies are widely used for healthcare decision-making in chronic conditions. Our aim was to assess the cost-of-illness of adult atopic dermatitis (AD) from the societal perspective in Hungary.

**Methods:**

We conducted a multicentre, cross-sectional questionnaire survey between February 2018 and January 2021. Data was collected from consecutive AD patients aged ≥ 18 years and their physicians at dermatology departments in Hungary. We calculated direct and indirect costs, including costs for treatments, outpatient visits, hospital admissions, informal care, travel costs and productivity loss. To assess indirect costs, the Work Productivity and Activity Impairment (WPAI) questionnaire was used to collect data, and costs were estimated with the human capital approach. Generalized linear model was used to analyse predictors of total, direct and indirect costs.

**Results:**

Altogether 218 patients completed the survey (57.8% female) with an average age of 31.3 (SD = 11.7). Patients’ average Dermatology Life Quality Index (DLQI) score was 13.5 (SD = 8.5). According to Eczema Area and Severity Index (EASI) score, 2.3% (n = 5), 21.2% (n = 46), 54.4% (n = 118) and 22.1% (n = 48) had clear, mild, moderate, and severe AD, respectively. We found that the average total, direct medical, direct non-medical and indirect annual costs per patients were €4,331, €1,136, €747, and €2450, respectively, with absenteeism and presenteeism being the main cost drivers, accounting for 24% and 29% of the total cost of AD. A one-year longer disease duration led to, on average, 1.6%, and 4.2% increase in total and direct non-medical costs, respectively. Patients with worse health-related quality of life (higher DLQI score) had significantly higher total, direct medical, direct non-medical costs, and indirect costs.

**Conclusions:**

Our results indicate a substantial economic burden of AD from a societal perspective, mainly driven by productivity losses.

**Supplementary Information:**

The online version contains supplementary material available at 10.1186/s12913-023-09840-7.

## Background

Cost-of-illness (COI) studies are widely used in healthcare decision-making for chronic conditions [1]. Atopic dermatitis (AD) is a chronic inflammatory skin condition with a worldwide prevalence of approximately 15–20% among children and 1–3% among adults [2]. In the United States, the estimated prevalence of this condition in adults ranges from 7 to 11% [3]. The prevalence of childhood AD has shown an increasing tendency, thereby contributing to an increasing public health burden [4]. AD significantly impacts a patient’s health-related quality of life (HRQoL) and results in a substantial burden of both direct and indirect costs on both an individual and societal level [4–10]. A recent review highlights that economic evaluations for AD may be needed in order to better understand the value of new treatments [11]. According to a global study on disease burden, a significant portion of the total burden measured in disability adjusted life-years (DALYs) in the United States is attributed to dermatitis, including atopic, contact and seborrheic dermatitis [12].

The annual costs of AD in the United States were estimated to be $US 5.297 billion in 2015 for the patient population [5]. The annual direct cost per patient of AD in the Asia-Pacific region ranged from $US199 in Thailand to $US1,250 in South Korea [5, 10]. In Europe, the annual societal costs were estimated to be EUR 30 billion, with half of the total costs attributed to productivity losses [13]. In a recent German study, the total annual cost per patient was found to be EUR 3,616 [14]. Similarly, a register-based study conducted in Denmark estimated the mean healthcare cost per individual to be EUR 4,930 [15].

AD can significantly impact patients’ ability to work, indicating that indirect costs associated with AD may be substantial [16]. A comprehensive European study found that 57% of patients with AD missed at least one day of work in the previous year due to their skin condition [17].

Estimating the cost of illness associated with AD in Hungary has the potential to provide valuable input data for resource allocation and health policy decisions. COI analyses have been conducted worldwide over the past two decades to assess the economic burden of AD. However, cost data from Hungary is still lacking for adult patients, as previous studies have only reported cost data for children [18]. Moreover, the transferability of costs from international studies is severely limited [19], highlighting the need for country-specific cost data to inform healthcare policy and resource allocation decisions.

We adopted a societal perspective for our calculation of COI in order to include all cost items that are relevant to society. This includes direct medical costs, direct non-medical costs and indirect costs, which allows for a comprehensive analysis of all the opportunity costs associated with a disease. We aimed to measure the resource and costs related to AD and analyse the main cost drivers from the perspective of Hungarian society.

## Methods

### Study sample

A cross-sectional study was conducted at two academic dermatology departments and one dermatology outpatient clinic (in different regions and type of settlements) in Hungary between February 2018 and January 2021 [20, 21]. Consecutive patients who were over 18 years of age and diagnosed with AD were enrolled. Our study was performed in compliance with the ethical standards set by the National Scientific and Ethical Committee (reference number: 29,655/2018/EKU) and in accordance with the 1964 Helsinki Declaration. All participants provided their informed consent.

The first part of the questionnaire was completed by patients and involved questions related to demographic characteristics, employment status, HRQoL and utilisation of healthcare services in the past 12 months. General HRQoL was assessed by employing the EQ-5D-5L, the EQ visual analogue scale (EQ VAS) [22, 23], and the Dermatology Life Quality Index (DLQI). The EQ-5D questionnaire is the most commonly used generic preference-weighted tool to measure HRQoL in dermatology. It has demonstrated good validity in several dermatological conditions, including AD [20, 21, 24–27]. The EQ-5D-5L version has five different levels of problems in the five dimensions (mobility, self-care, usual activities, pain/discomfort, anxiety/depression), resulting in 3125 different health states [22]. The EQ-5D-5L questionnaire comprises a visual analogue scale known as the EQ-VAS, which allows respondents to rate their current health status on a scale of 0 (representing the worst imaginable health) to 100 (representing the best imaginable health). Each EQ-5D-5L health profile can be assigned an index score, which we used the country-specific value set for Hungary [23].

The DLQI questionnaire is a dermatology-specific self-reported questionnaire [11] containing ten items that cover the common issues that affect the HRQoL of patients with skin diseases. Each item on the DLQI is scored on a 4-point scale: ‘not at all’ or ‘not relevant’ = 0, ‘a little’ = 1, ‘a lot’ = 2 and ‘very much’ = 3. The total DLQI score is calculated by summing up the scores of all questions, resulting in a maximum score of 30 and a minimum score of 0. A higher score indicates a greater impairment in the patient’s HRQoL. The bands used to categorise DLQI scores are as follows: 0–1 no effect on the patient’s life, 2–5 indicating a small effect, 6–10 indicating a moderate effect, 11–20 indicating a very large effect and 21–30 indicating an extremely large effect [28].

To measure absenteeism and presenteeism, we used the Work Productivity and Activity Impairment Questionnaire Specific Health Problem (WPAI-SHP) [22], in which patients were asked about their employment status and hours mi ssed from work during the past week. Patients had to first indicate whether they were currently doing paid work (Q1). The following section (Q2-Q5) is only relevant for participants who are employed and includes questions about the number of hours missed from work due to health reasons (Q2) and other reasons (Q3). Respondents were required to indicate the number of hours they worked in the past seven days (Q4). The questionnaire measures the extent of labour productivity loss experienced at work on an 11-point scale, ranging from 0 (not affected) to 10 (completely prevented). The last question (Q6) pertains to the degree to which the patient’s health issues impacted their daily activities. This question uses an 11-point rating scale (0: not affected, 10: completely prevented). WPAI scores are expressed as a percentage, where higher values indicate greater limitations and loss of productivity [22, 23].

The second part of the questionnaire was completed by dermatologists. Based on the medical records provided, they presented data on the clinical characteristics and treatments administered in the last 12 months. Disease severity was assessed using the Eczema Area and Severity Index (EASI) score [28]. The cut-off values for disease severity were as follows: 0 for clear, 0.1–5.9 for mild, 6.0–22.9 for moderate and 23.0–72 for severe, as reported by Chopra et al. (2017) [29].

### Cost calculation

A prevalence-based cost analysis was conducted from a societal perspective using a bottom-up approach. We followed the methods used in previous studies on the cost of illness in dermatology [30, 31]. Our analysis included direct medical costs, direct non-medical costs (such as informal care and travel) and indirect costs related to productivity loss. All costs in this study were based on price levels from 2020 and reported in euros (€1 = 351.17 HUF) [32]. The cost analysis was performed in two steps. First, all resources consumed by each patient were identified. In the second step, the unit costs of resources were multiplied by the quantities used. Unit costs for all identified resources were obtained from official published sources, including the National Health Insurance Fund of Hungary (NHIFH) and the Hungarian Central Statistical Office. The unit costs are specified in Supplementary Table 1.

### Measuring resource use

We used patient-level data on healthcare utilisation, informal care, out-of-pocket payments and productivity. The recall period varied depending on the frequency of resource use: general practitioner (GP) visits were recorded for the previous month, outpatient specialist visits for the previous three months and hospitalisations for the previous year.

Both reimbursed and unreimbursed healthcare resource utilisation was recorded in our questionnaire. Reimbursed services and products, such as healthcare visits at different levels of care (GP, dermatologist and other outpatient visits), home medical care, inpatient care, ambulance transportation and medication use related to AD, were considered. Other cost items, such as ambulance transportation and home medical care services related to AD, were also taken into consideration. Out-of-pocket payments for non-reimbursed medical services and products, such as private dermatologist consultations, over-the-counter medications and other medicinal products, were recorded.

Direct non-medical resource use, such as travel costs and informal care, was also collected. To estimate travel expenses, patients were asked to specify their mode of transportation to the healthcare provider and the distance between their residence and the clinic. Patients also reported the number of weekly hours they received for paid and informal care.

Indirect costs were also considered in this study, including absence from work (absenteeism), reduced productivity at work (presenteeism) and disability. Productivity losses were estimated based on the answers provided in the WPAI questionnaire. As the percentage of patients receiving inpatient care at the time of the survey was high (40.4%, n = 88), we took a more conservative approach to calculating their indirect costs. We multiplied their responses in the WPAI questionnaire by the number of hospital visits they reported per year, including an additional occasion for when they completed the survey while hospitalised.

### Valuing units of resources

For visits to GPs and outpatient specialists, we used the average costs reported by the NHIFH, which were €6.0 and €8.4, respectively [33]. The cost of hospital admissions related to AD was valued at €438.3, based on the payer tariff for the Diagnosis Related Group for ‘severe skin disease’ [34]. The cost of medications was estimated using the pharmaceutical reimbursement price list of the National Health Insurance Fund as well as the retail prices for non-reimbursed products [35]. The cost of ambulance transportation is calculated by multiplying the distance between the patient’s home and the clinic by the cost per kilometre (€0.1). We also took into account other transportation expenses by using the official public transportation tariffs (for trains and long-distance buses) or official fuel consumption data (for private cars). The cost of home remodelling and lifestyle changes was self-reported by the patient. The monetary value of informal care was estimated by calculating the opportunity cost of care time based on the average net hourly wage of €4.4 [36]. All components of productivity loss, including absenteeism, presenteeism and disability pension, were valued using the human capital approach. The average gross hourly wage levels in Hungary (€7.6) were used for this valuation [36]. A list of unit costs and their respective data sources is available in Supplementary Table 1.

### Statistical analysis

Statistical analyses were conducted using SPSS 27 (Armonk, NY: IBM Corp.). Our cost data was not normally distributed and was highly skewed to the right. Therefore, we utilised bootstrap testing with 95% confidence intervals and accelerated bias correction. We performed cost comparisons between groups based on disease severity, DLQI bands and sex using bootstrap t-tests and one-way bootstrap ANOVA with post hoc Bonferroni tests. Additionally, we assessed the relationship between age, disease duration and costs using Spearman’s rank correlation analysis. We constructed a multivariate generalised linear model to investigate the correlation between patients’ socio-demographic and clinical characteristics and their direct medical, direct non-medical, indirect and total costs. The explanatory variables and factors included age, sex, disease duration and disease severity measured by EASI score, DLQI and EQ-5D-5L index scores, and a gamma distribution with a log-link function was applied. All regression coefficients were exponentiated to facilitate interpretation. All statistical tests were conducted as two-sided tests, and the results were deemed statistically significant if the p-values were less than or equal to 0.05.

## Results

### Characteristics of the patient population

Altogether, 218 patients completed our survey, of whom 57.8% were female, with an average age of 31.3 years (18.0–73.0). The vast majority of respondents had at least a secondary education (94.4%). Half (50.0%) of the respondents were employed full-time, while 0.9% (n = 2) were disability pensioners due to AD.

In our sample, the majority of patients (89.0%) had comorbidities. The most prevalent conditions were rhinitis allergica (59.2%), pollen allergy (48.6%), dust allergy (36.7%), asthma bronchiale (33.9%) and any other dermatological diseases (16.1%). The average duration of the disease was 19.02 (SD = 12.91) years. One-third of the patients (33.9%) had a family history of AD, as shown in Table 1. More than half of the patients (59.6%, n = 130) were receiving outpatient care at the time of the survey, while 40.4% (n = 88) were receiving inpatient care. A total of 159 patients received systemic treatment, of whom 32 were taking systemic steroids, 21 were taking cyclosporin, 11 were taking methotrexate and eight were taking dupilumab (five of whom were taking it at the time of the survey). Only three patients were receiving acyclovir.

**Table 1:** General and sociodemographic characteristics of the sample

	Mean (SD) or N (%)
Age (years)	31.3 (11.7)
Sex	
*Female*	126 (57.8%)
*Male*	92 (42.2%)
Education (missing = 2)	
*Primary*	12 (5.6%)
*Secondary*	112 (51.9%)
*Tertiary*	92 (42.6%)
Employment	
*Employed full-time*	109 (50.0%)
*Employed part time*	24 (11.0%)
*Retired*	7 (3.2%)
*Disability pensioner*	6 (2.8%)
*Unemployed*	12 (5.5%)
*Student*	60 (27.5%)
*Other*	23 (10.6%)
Disease duration (years) (missing = 3)	19.02 (12.91)
AD in the family	74 (33.9%)
Having comorbidities	194 (89.0%)
Dermatologic	35 (16.1%)
Non-dermatologic	194 (89.0%)
Number of comorbidities	3.39 (2.35)
None	24 (11.0%)
1–2	63 (28.9%)
3–4	61 (28.0%)
5–6	48 (22.0%)
≥ 7	22 (10.1%)
Disease severity (EASI score)	
Clear (0)	5 (2.3%)
Mild (0.1–5.9)	46 (21.2%)
Moderate (6.0–22.9)	118 (54.4%)
Severe (23.0–72)	48 (22.1%)
Disease severity VAS (patient reported) (0–10)	6.04 (2.74)
DLQI (0–30)	13.45 (8.46)
EQ-5D-5L index (-0.848-1)	0.82 (0.22)
EQ VAS (0-100)	69.15 (20.50)

The average DLQI score of the patients was 13.45 (SD = 8.46). According to the EASI score, 2.3% (n = 5), 21.2% (n = 46), 54.4% (n = 118) and 22.1% (n = 48) had clear, mild, moderate, and severe AD, respectively.

### Resource use

**Fig. 1:**
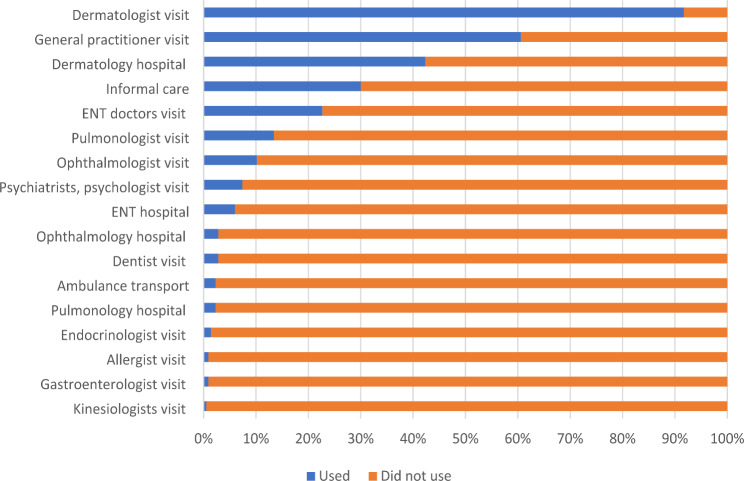
Resource utilization due to AD in the past 12 months *ENT = Diseases of the ear, nose, and throat

Patients had an average of 10.5 (SD = 18.3) consultations with their GP and 10.0 (SD = 8.2) consultations with a dermatologist annually for the treatment of AD. The mean number of hospital admissions due to dermatological issues per year was 0.78 (SD = 1.74). The most frequently used medical services were visits to a dermatologist, a GP, and admission to a dermatological hospital (91.7%, 61.6% and 42.3%, respectively) (Fig. 1). The mean hours of informal care received per week were 3.08 (SD = 9.85) (Supplementary Table 2). On average, patients missed 4.5 (11.5) hours of work per week. Patients with mild, moderate and severe disease, as determined by their EASI score, missed 1.2 (3.6), 3.3 (9.4) and 11.5 (17.8) hours of work, respectively (p = 0.004). Patients in the EASI ‘clear’ category did not report any productivity losses.

### Costs of atopic dermatitis

The mean annual total cost per patient ranged from €0 to €29,783, with an average cost of €4,331. One patient had no cost at all. Direct medical costs (€1,136), direct non-medical costs (€747) and indirect costs (€2,450) accounted for 27%, 17% and 56% of the total costs, respectively (Table 2). There was no statistically significant difference in the mean total costs between male and female patients (€4,263 vs. €4,380, p = 0.597).

**Table 2:** Average direct and indirect costs per patient per year (2020, EUR)*

	Mean	Median	Minimum	Maximum	CI95% lower bound	CI 95% upper bound
General practitioner visit	58	0	0	312	46	71
Outpatient visit	117	101	0	774	103	131
Hospital admission	396	0	0	5,260	288	503
Treatment costs						
Systemic treatments	88	0	0	2,710	44	132
Antihystamines and asthma medication**	78	17	0	1,849	55	101
Antibiotics	18	0	0	220	13	24
Topical treatments	5	2	0	42	4	6
Phototerapy	0.1	0	0	8.9	0	0.14
Non reimbursed products	148	77	0	1,401	121	174
Non reimbursed medical services (e.g. private physician)	232	0	0	3,417	166	298
***Direct medical***	***1,136***	***695***	***0***	***6,391***	***988***	***1,284***
Travel	5	4	0	34	4	6
Ambulance transport	1.2	0	0	177	0	2.9
Home remodelling	40	0	0	2,848	9	71
Lifestyle change***	49	0	0	1,566	26	73
Informal care	637	0	0	12,848	404	870
Paid carer	22	0	0	3,417	0	54
***Direct non-medical***	***747***	***22***	***0***	***12,946***	***509***	***984***
***Direct costs***	***1,881***	***933***	***0***	***17,080***	***1,569***	***2,193***
Absenteeism	1,047	0	0	23,712	578	1,515
Presenteeism	1,262	0	0	21,341	868	1,655
Permanent disability (n = 2)	142	0	0	15,443	0	339
***Indirect costs***	***2,450***	***0***	***0***	***23,712***	***1,783***	***3,117***
***Total costs***	***4,331***	***2,000***	***0***	***29,783***	***3,561***	***5,110***

* Figures may not add up within categories due to rounding

**Due to the symptoms of AD

*** Non-medical products and services purchased due to AD

**Fig. 2:**
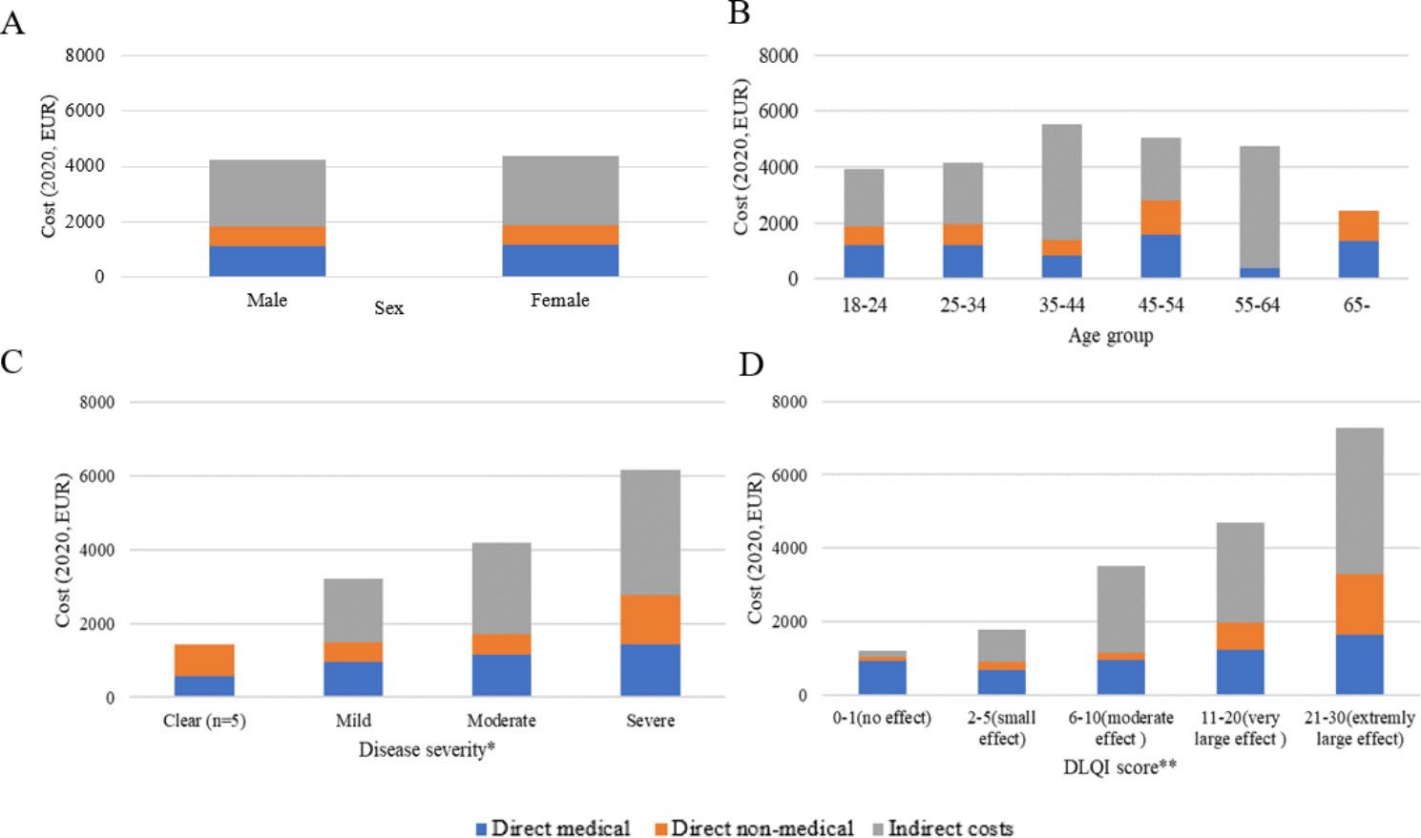
Per patient costs by cost categories in subgroups of patients (2020, EUR) *Disease severity expressed withthe Eczema Area and Severity Index (EASI) score banding according to Chopra et al. (2017) [10] **DLQI = Dermatology Life Quality Index; banding system for DLQI scores: Hongbo et al. (2005) [28]

The mean annual total costs for patients with clear, mild, moderate, severe and very severe AD, as determined by the EASI score, were €1,442, €3,205, €4,178 and €6,158, respectively (p = 0.014). Patients in the ‘small effect’ DLQI band (€1,785) had lower total costs than those in the ‘moderate’ (€3,500), ‘very large’ (€4,685) or ‘extremely large’ (€7,281) effect bands (p = 0.001) (Fig. 2). There was no significant correlation between age (p = 0.162) and total costs. However, a very weak positive correlation was observed between disease duration (r = 0.163, p = 0.017) and total costs.

### Productivity losses

The mean annual costs for absenteeism and presenteeism were €1,047 (SD=€3,510) and €1,262 (SD=€2,951), respectively. The average number of work hours missed per patient per year was 5.8 days, and an additional 6.9 days were lost due to presenteeism. Two patients in our sample were receiving disability pensions due to AD.

### Regression analysis

We found two significant explanatory variables in our models: higher DLQI score and longer disease duration was associated with higher average cost. DLQI score was found to be a significant determinant of the total, direct medical, direct non-medical costs, and indirect costs showing a positive association with each, 1-point increase in DLQI score led to. on average 5.1%, 2.3%, 11.3% and 4.7% increase respectively. Disease duration was a significant determinant of the total, and direct non-medical costs: 1-year increase in disease duration led to, on average, 1.6%, and 4.2% increase, respectively. We found no significant differences by sex, age, or EASI score in either model (p > 0.05).

Disease duration was a significant determinant of the total, and direct non-medical costs, 1-year increase in disease duration led to, on average, 1.6%, and 4.2% increase, respectively. The DLQI score was a significant determinant of the total, direct medical, direct non-medical costs, and indirect costs showing a positive association with each. Regarding indirect costs, we found no significant differences by sex, age, or disease duration (p > 0.05). (Table 3).

**Table 3:** Determinants of total, direct and indirect costs per patient per year in subgroups of patients (2020, EUR)

	Total cost		Direct medical cost		Direct non-medical		Indirect cost	
Parameter	Expβ*	p-value	Expβ*	p-value	Expβ*	p-value	Expβ*	p-value
Female gender	1.0938	0.5680	0.9805	0.8845	0.9900	0.9707	0.9510	0.8570
Age	0.9991	0.9010	1.0020	0.7267	1.0005	0.9563	0.9978	0.8978
Disease duration	**1.0159**	**0.0084**	0.9945	0.2610	**1.0424**	**0.0005**	1.0128	0.2950
DLQI score	**1.0509**	**< 0.0001**	**1.0228**	**0.0060**	**1.1127**	**< 0.0001**	**1.0468**	**0.0247**
EASI score	1.0106	0.1572	1.0106	0.0667	0.9984	0.8921	1.0014	0.9219
Maximum likelihood estimate (Scale)	3.1582		2.1191		24.2642		6.4366	
Goodness of fit Deviance/df	1.389		0.865		4.500		2.485	

*Model coefficients were exponentiated, significant variables’ Expβ and p values are formatted “bold”

## Discussion

In our current study, we estimated the costs of adult AD in Hungary. Our analysis was based on a cross-sectional survey conducted at two university dermatology clinics and one dermatology outpatient clinic in Hungary, with a total of 218 patients and their physicians recruited. The costs associated with adult AD were €4,331 per patient per year.

We found a significant difference in total costs, direct medical costs, direct non-medical costs and indirect costs based on the DLQI score, indicating that impairments in HRQoL may be important factors in determining resource utilisation. Disease duration was a significant determinant of both total and direct non-medical costs. Direct non-medical costs were mainly attributed to informal care costs (85%). We also found a weak but significant positive correlation (r = 0.160, p < 0.019) between the burden of informal care and disease duration, suggesting that care needs may increase as the disease progresses. Although it was not a significant explanatory factor in the regression, it should be mentioned that there was a significant difference between the EASI categories. Patients with more severe symptoms had a higher average cost (p = 0.014). The mean annual total costs ranged from €1,442 (clear) to €6,158 (very severe) as per the EASI groups.

Extensive literature on COI studies is available for various dermatological conditions, and several studies on disease burden and cost of illness have been conducted worldwide for AD [11]. In a cross-sectional survey of Japanese physicians, the mean annual expected cost per patient was JPY 136,501 [37]. According to an analysis of a claims database in the United States, the adjusted total costs ranged from $3,302 per patient per year for less severe cases to $4,463 for more severe cases [38]. In a German study, the total costs for patients with mild AD were €1,466 per person per year, while patients with moderate-to-severe AD had total costs of €5229, which is consistent with our current findings [14]. In a multicentre observational study conducted in Italy, the total annual burden of AD was found to be €4,284 per patient, which is similar to our current findings. The study also reported that 60.8% of the costs were attributed to productivity losses, which is slightly higher than our result of 56.6% [39]. According to an observational cohort study conducted in the Netherlands, the total direct costs amounted to €15,231, which is higher than our current findings [40]. In a separate French study, the mean annual out-of-pocket cost for severe AD was €462.1 and €247.4 for moderate AD, which is comparable to our results [41].

In our study, the average direct healthcare cost per patient was €1,154 annually. Hospital admissions (€396), non-reimbursed services (€232) and non-reimbursed products (€148) were identified as the largest cost drivers. It is important to note that the costs of non-reimbursed products and services were self-reported by the patients. In a study conducted in the United States, the median annual out-of-pocket expense was US$600 (with a range of US $0–$200,000), which is higher than the findings of our current study [42].

Direct non-healthcare costs amounted to €727 per patient per year, with the cost of informal care being the main cost driver at €637. Almost one-third (30%) of the patients reported receiving informal care, which is relatively high considering the young age of the patients in our sample (average age was 31.34 years (SD = 11.68)). The proportion of informal care recipients was similar to that reported for hidradenitis suppurativa (25.0%) and pemphigus (25.7%) in recent Hungarian studies [24, 30].

As AD often affects working-age adults, the indirect costs may be a significant component of the total cost of illness associated with AD in adults. In our current study, indirect costs accounted for €2,450, which represents 56.6% of the total costs. On average, patients missed 2.7 h of work per week, and half of them (50.0%) were employed full-time. Barbeau et al. (2006) analysed the burden of AD in Canada and reported that the cost of absenteeism per patient increased with disease severity. In addition, patients lost 9.5 h annually due to AD on average [43], which is higher than the average in our sample.

In our current study, we found that the average total cost of the dermatological condition we examined in Hungary was slightly higher than that of pemphigus (€4,331 vs. €3,995, with direct costs accounting for 42%) [30]. However, it was lower than the cost of treating hidradenitis suppurativa (€6,791, with direct costs accounting for 47%) [24] and psoriasis, where the mean total cost was €9,254 per patient per year, with direct costs accounting for 86% of the total costs [44]. The average age varied greatly across the four samples: 57 years in pemphigus [30], 37 years in hidradenitis suppurativa [24], 51 years in psoriasis [44] and 31 years in our current sample.

Our current results may provide valuable information for making decisions related to health policy decision making. New biological drugs, such as upadaticinib undergoing clinical testing [45] and lebrikizumab under evaluation by the EMA, are emerging in the market. Meanwhile, some drugs like abrocitinib have recently received approval [46, 47]. Therefore, a decision on their financing must be made soon, for which detailed knowledge of costs, preferably from a societal perspective, is required. Adopting a societal perspective in economic evaluation enables the inclusion of cost items that are relevant to society. Several national guidelines (Norway, Denmark, Italy, France, Germany, Poland, and Slovakia) require that the analysis be conducted from a societal perspective. In Hungary, it is recommended to supplement the base case health care perspective with results calculated from a societal perspective [48, 49]. Ignoring costs that are not included in the healthcare budget could potentially affect the findings of economic evaluations [46]. The high proportion of indirect costs, which accounted for more than half of the total costs, highlights the need for intervention programmes aimed at enhancing work productivity in patients with AD. This is particularly important given that the majority of patients in our sample were of working age. We estimated very high presenteeism costs as well, which suggests that AD may often be associated with reduced work productivity while on the job. Our study presents COI data from a societal perspective for adult patients with AD in Hungary. The aim is to provide healthcare policymakers with valuable information to make informed decisions regarding priority setting and resource allocation. The cost of AD can also be compared with that of other conditions to inform policy planning in the fields of health and social care.

Our current study has several limitations. This study was primarily conducted in academic dermatology departments, which may introduce selection bias and limit the representativeness of our sample to the border population of Hungarian patients with AD. The data were collected retrospectively through self-completed questionnaires, which may introduce recall bias. Furthermore, since a significant number of respondents received inpatient care at the time of the study, period, it is possible that the indirect costs have been overestimated. Considering that the care for AD in Hungary has undergone significant changes since our data collection, such as the increased use of dupilumab among patients, further research may be necessary.

## Conclusion

We analysed the costs associated with adult AD in Hungary and found significant expenses for both the healthcare system and society. The costs of AD were found to be comparable to those of other chronic skin diseases, which is consistent with international findings. Indirect costs exceeded direct costs (€2,450 vs. €1,881). More severe clinical symptoms, as measured by the EASI score, and worse self-reported quality of life related to skin, as measured by the DLQI score, were found to be associated with higher costs. Given the lack of COI data for adult AD patients in Hungary, our results offer valuable insights into resource utilisation and cost inputs that can be used for conducting cost-effectiveness analyses, especially for newly adopted biological treatments.

### Electronic supplementary material

Below is the link to the electronic supplementary material.

**Supplementary Table 1** Listing of units of resources included in cost analysis and unit costs. **Supplementary Table 2**. Resource utilization

## Data Availability

All data for this study are available from the corresponding author upon reasonable request.
