# Supplementary material for: Societal costs and health related quality of life in adult atopic dermatitis
Source: BMC Health Serv Res. 2023 Aug 14;23:859. doi: 10.1186/s12913-023-09840-7 (PMC10426091; doi:10.1186/s12913-023-09840-7)
Supplement: Supplementary file 1 — Supplementary Table 1 Listing of units of resources included in cost analysis and unit costs. Supplementary Table 2. Resource utilization [file 12913_2023_9840_MOESM1_ESM.docx]

**Supplementary Table 1 Listing of units of resources included in cost analysis and unit costs**

| **Resources** | **Unit cost** | **Source** |
| --- | --- | --- |
| **Treatments** | |  |
| Aciclovir | 0.00084€/mg | Official price list of the National Health Insurance Fund of Hungary ^1^ Online drug database^2^ |
| Amoxicillin (Aktil Duo) | 0.00050€/mg |  |
| Amoxicillin (Augmentin) | 0.00064€/mg |  |
| Azathioprine | 0.00287€/mg |  |
| Azithromycin | 0.00234€/mg |  |
| Azitromicin | 0.00188€/mg |  |
| Betamethasone | 0.37759€/mg |  |
| Bilastine | 0.02736€/mg |  |
| Budesonide | 0.04748€/mg |  |
| Cefalosporin | 0.00140€/mg |  |
| Cefixime | 0.00462€/mg |  |
| Cefuroxime | 0.01730€/mg |  |
| Cefuroxime (Zinnat) | 0.00131€/mg |  |
| Ceroxim | 0.00077€/mg |  |
| Cetirizin | 0.00085€/mg |  |
| Chloropyramine | 0.00368€/mg |  |
| Ciprofloxacin | 0.00056€/mg |  |
| Clindamycin | 0.00085€/mg |  |
| Corticosteroid (systemic) | 0.02690 €/mg |  |
| Cyclosporine | 0.02475€/mg |  |
| Desloratadine (Lordestin) | 0.01851€/mg |  |
| Desloratadine (Aerius) | 0.03222€/mg |  |
| Doxycylin | 0.00069€/mg |  |
| Fexofenadine | 0.00096€/mg |  |
| Fexofenadin-hidroklorid | 0.00246€/mg |  |
| Fluticasone | 0.00099€/mg |  |
| Isoconazole | 0.2650€/g |  |
| Levocetirizin | 0.04473€/mg |  |
| Loratadine | 0.00917€/mg |  |
| Methotrexate | 0.05644€/mg |  |
| Montelukast | 0.01299€/mg |  |
| Mupirocin | 0.3650€/mg |  |
| Salbutamol | 0.01236€/mg |  |
| Theophylline | 0.00024€/mg |  |
| **Healthcare services** | | |
| Ambulance transportation | 0.1300 €/km | National Health Insurance Fund of Hungary ^4,5^ |
| Outpatient specialist visit | 8.42 €/visit | National Health Insurance Fund of Hungary ^4,5^ |
| GP visit | 5.99 €/visit | National Health Insurance Fund of Hungary ^4,5^ |
| Hospital admission | 438.33€/admission | National Health Insurance Fund of Hungary ^4,5^ |
| Private outpatient specialist visit | given by the patient | not applicable |
| **Transportation costs** | | |
| Travel by car | 0.1304 €/km | National Tax and Custom Administration^6^ |
| Travel by long distance bus | 0.0648€/km | Volánbusz Transport Company^7^ |
| Travel by train | 0.0536 €/km | MAV Group^8^ |
| Local public transportation (bus, tram, metro) | 2.0 €/occasion | BKK^9^, DKV^10^ |
| **Productivity loss and informal care** | | |
| Missed working hours | 7.6 €/hour | Hungarian Central Statistical Office^12,13^ |
| Informal care hours | 4.4 €/hour |  |

1:http://www.neak.gov.hu/felso_menu/szakmai_oldalak/gyogyszer_segedeszkoz_gyogyfurdo_tamogatas/egeszsegugyi_vallalkozasoknak/pupha/Vegleges_PUPHA.html Accessed: 15. 02.2021

2: https://www.hazipatika.com/ Accessed: 15.01.2021

3: 9/1993. (IV. 2.) NM rendelet az egészségügyi szakellátás társadalombiztosítási finanszírozásának egyes kérdéseiről Hatályos: 2017.04.01-től.<http://www.hbcs.hu/images/2017/jo/9_1993.pdf> Accessed: 01.10.2020

4:http://www.neak.gov.hu/felso_menu/szakmai_oldalak/gyogyito_megeleozo_ellatas/adatbazisok/torzsek/torzsek Accessed: 10.01.2021.

5: Payments for health care services http://www.neak.gov.hu/felso_menu/szakmai_oldalak/gyogyito_megeleozo_ellatas/adatbazisok/torzsek/torzsek Accessed: 10.12.2020.

6: https://nav.gov.hu/ugyfeliranytu/uzemanyag/2022_uzemanyagar Accessed: 15.01.2021

7: <http://www.volanbusz.hu/hu/utazasi-informaciok/jegyvasarlas/dijszabas/dijszabas# mozTocId138916> Accessed: 15.01.2021

8: <https://www.mavcsoport.hu/mav-start/belfoldi-utazas/arak-es-kedvezmenyek/teljesaru-menetdijak-egy-utra> Accessed: 15.01.2021

9: <http://www.bkk.hu/tomegkozlekedes/jegyek-es-berletek/jegy-es-berletarak/> Accessed: 16.12.2020

10: <http://www.dkv.hu/jegyek_berletek_potdij> Accessed: 18. 12.2020

11: <http://szkt.hu/penztarak> Accessed: 16.12.2020

12: <http://www.ksh.hu/docs/hun/xftp/stattukor/munkaido.pdf> Accessed: 16.11.2020

13: https://www.ksh.hu/stadat_files/mun/hu/mun0046.html Accessed: 16.12.2021

**Supplementary Table 2. Resource utilization**

|  | N | Minimum | Maximum | Mean | Std. Deviation | Had at least 1 use (%) |
| --- | --- | --- | --- | --- | --- | --- |
| General practitioner visit | 213 | 0 | 52 | 10.54 | 18.26 | 39.4% |
| Dermatologist visit | 216 | 0 | 52 | 10.04 | 8.17 | 91.7% |
| ENT doctors visit | 216 | 0 | 18 | 1.48 | 4.37 | 22.7% |
| Pulmonologist visit | 216 | 0 | 24 | 0.67 | 2.18 | 13.4% |
| Ophthalmologist visit | 216 | 0 | 12 | 0.65 | 2.17 | 10.2% |
| Psychiatrist, psychologist visit | 216 | 0 | 16 | 0.78 | 3.06 | 7.4% |
| Endocrinology visit | 216 | 0 | 4 | 0.06 | 0.47 | 1.4% |
| Dentist visit* | 216 | 0 | 12 | 0.19 | 1.27 | 2.8% |
| Gastroenterologist visit | 216 | 0 | 4 | 0.04 | 0.38 | 0.9% |
| Allergist visit | 216 | 0 | 20 | 0.13 | 1.46 | 0.9% |
| Kinesiologist visit | 216 | 0 | 8 | 0.04 | 0.54 | 0.5% |
| Dermatology hospital | 215 | 0 | 14 | 0.78 | 1.74 | 42.3% |
| ENT hospital | 216 | 0 | 5 | 0.08 | 0.42 | 6.0% |
| Pulmonology hospital | 216 | 0 | 1 | 0.02 | 0.15 | 2.3% |
| Ophthalmology hospital | 216 | 0 | 2 | 0.03 | 0.20 | 2.8% |
| Psychiatry hospital | 216 | 0 | 0 | 0.00 | 0.00 | 0.0% |
| Ambulance transport | 218 | 0 | 4 | 0.04 | 0.30 | 2.3% |
| Informal care (weekly hour) | 214 | 0 | 56 | 3.08 | 9.85 | 30.0% |
| Absenteeism  (hours/year) | 218 | 0 | 3120 | 137.7 | 461.8 | 22.0% |
| Presenteeism  (hour/year) | 218 | 0 | 2880 | 165.9 | 388.3 | 39.9% |

*Due to atopic dermatitis
